# Cross-sectional imaging of the torso reveals occult injuries in asymptomatic blunt trauma patients

**DOI:** 10.1186/s13017-019-0287-5

**Published:** 2020-01-09

**Authors:** Gregory J. Roberts, Lewis E. Jacobson, Michelle M. Amaral, Courtney D. Jensen, Louis Cooke, Jacqueline F. Schultz, Alexander J. Kinstedt, Jonathan M. Saxe

**Affiliations:** 1 Trauma Department, St. Vincent Indianapolis Hospital, 8240 Naab Road #100, Indianapolis, IN 46260 USA; 2 Department of Economics, University of the Pacific, Stockton, CA USA

**Keywords:** Blunt trauma, Computed tomography (CT), Occult injury, Asymptomatic

## Abstract

**Background:**

High morbidity and mortality rates of trauma injuries make early detection and correct diagnosis crucial for increasing patient’s survival and quality of life after an injury. Improvements in technology have facilitated the rapid detection of injuries, especially with the use of computed tomography (CT). However, the increased use of CT imaging is not universally advocated for. Some advocate for the use of selective CT imaging, especially in cases where the severity of the injury is low. The purpose of this study is to review the CT indications, findings, and complications in patients with low Injury Severity Scores (ISS) to determine the utility of torso CT in this patient cohort.

**Methods:**

A retrospective review of non-intubated, adult blunt trauma patients with an initial GCS of 14 or 15 evaluated in an ACS verified level 1 trauma center from July 2012 to June 2015 was performed. Data was obtained from the hospital’s trauma registry and chart review, with the following data included: age, sex, injury type, ISS, physical exam findings, all injuries recorded, injuries detected by torso CT, missed injuries, and complications. The statistical tests conducted in the analysis of the collected data were chi-squared, Fischer exact test, and ANOVA analysis.

**Results:**

There were 2306 patients included in this study, with a mean ISS of 8. For patients with a normal chest exam that had a chest CT, 15% were found to have an occult chest injury. In patients with a negative chest exam and negative chest X-ray, 35% had occult injuries detected on chest CT. For patients with a negative abdominal exam and CT abdomen and pelvis, 16% were found to have an occult injury on CT. Lastly, 25% of patients with normal chest, abdomen, and pelvis exams with chest, abdomen, and pelvis CT scans demonstrated occult injuries. Asymptomatic patients with a negative CT had a length of stay 1 day less than patients without a corresponding CT. No incidents of contrast-induced complications were recorded.

**Conclusions:**

A negative physical exam combined with a normal chest X-ray does not rule out the presence of occult injuries and the need for torso imaging. In blunt trauma patients with normal sensorium, physical exam and chest X-ray, the practice of obtaining cross-sectional imaging appears beneficial by increasing the accuracy of total injury burden and decreasing the length of stay.

## Background

Trauma is the number one cause of death for people aged 1–44 and accounts for 19.2% of years of potential life lost in the United States. Additionally, the medical and loss-of-work cost from traumatic injuries totals well above $500 billion annually in the United States [1]. Given the magnitude of this problem, it is imperative that practitioners intervene in both a life-saving and cost-effective manner.

There are multiple ways to evaluate and treat trauma patients. Advanced trauma life support (ATLS) directs a rapid assessment of the acutely injured patient using physical examination, plain radiographs, and ultrasound to increase survival [2]. The use of torso computed tomography (CT) comes with vague recommendations. The ATLS guidelines do not detail recommendations as to the appropriate use of CT, and it is unclear which patients require this scan. Despite this, torso CT use for trauma patients has become much more common.

As CT technology has improved, more injuries are detected in shorter periods of time [3, 4]. This has led some centers to use CT scanning of the torso liberally [5–9]. Whereas others advocate for use in selected patients [10, 11]. Advocates of selective CT for trauma argue that the benefits do not outweigh the complications, which include IV contrast issues, radiation exposure, and cost [12–14].

The use of either torso CT or a “pan CT,” which includes CT of the head, cervical spine, chest, abdomen, and pelvis, has been shown to be beneficial in severely injured patients that do not have a reliable physical exam [5, 15–17]. However, even in evaluable patients, the sensitivity of physical examination and plain radiographs remain deadened to detecting some injuries, and there is controversy with regard to selecting the appropriate patients to undergo torso CT [18–22].

The use of pan CT in trauma for stable, unevaluable adult trauma patients is popular. The role of pan CT in the awake, mildly injured, evaluable patient is less clear and still widely debated [17, 20, 22]. Similar statements are true for a torso or thoracoabdominal CT [23].

In our center, both the Emergency Department (ED) and trauma attending physicians are involved in the initial workup of trauma patients, depending on the level of activation. This has led to a wide practice variation in which patients receive a torso CT. It is up to the discretion of the ED physician based upon the physical examination whether the patient will receive a scan. The purpose of this study is to review the CT indications, findings, and complications in patients with low Injury Severity Score (ISS) to determine the utility of torso CT in this patient cohort.

## Methods

A retrospective review of non-intubated, blunt trauma patients aged 15 years or older with an initial Glasgow Coma Scale (GCS) score of 14 or 15 evaluated in an American College of Surgeons verified level 1 trauma center from July 2012 to June 2015 was performed. Data was obtained from the trauma registry and chart review and included: age, sex, injury type, mechanism, ISS, physical exam findings, all injuries recorded, injuries detected by torso CT, missed injuries, and complications. The Institutional Review Board at St. Vincent Hospital granted permission for this study.

Physical exam (PE) findings were captured from trauma or ED notes, and all patients were seen by the ED attending physician. All trauma consults and code 1 activations were seen by the attending trauma surgeon. Physical exam findings were regularly recorded on a template trauma history and physical (H&P) form and were ‘visible trauma (location),’ ‘chest wall (CW) tenderness to palpation,’ ‘CW crepitus,’ ‘CW ecchymosis,’ ‘abdominal (Abd) ecchymosis,’ ‘Abd distension,’ ‘Abd tenderness,’ ‘flank ecchymosis.’

Recorded laboratory values included hemoglobin, international normalized ratio (INR), pH, lactate, base deficit, blood alcohol level, urine drug screen. Initial chest X-ray (CXR) and pelvic X-ray, if performed, were recorded. Initial chest/abdomen/pelvis (C/A/P) CTs were recorded, as well as delayed C/A/P CTs. All injuries and incidental findings were recorded. Delayed CT was defined as imaging performed after the initial evaluation in the ED. Repeat CTs for other reasons (re-evaluation, operative planning) were not recorded as such. Statistical tests conducted in the analysis of the collected data were chi-squared, Fischer exact test, and ANOVA analysis.

## Results

**Fig. 1:**
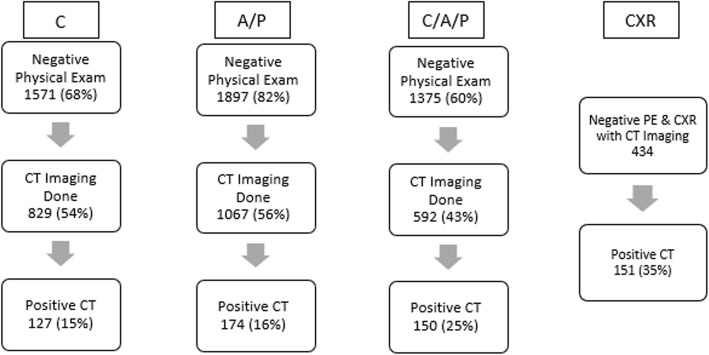
Diagram of the 3 main subgroups and chest X-ray (CXR) data

There were 2306 patients determined to be eligible for review from the registry. The mean ISS was 8, and the initial chest physical exam was normal in 1571 (68% of the patient population). The results are best broken up into three subgroups. Each of these subgroups has a negative physical exam in either the chest (C), abdomen and pelvis, (A/P), or chest, abdomen, and pelvis (C/A/P). In the first group, 829 (54%) of these patients received a chest CT, and 127 (15%) of these patients were found to have an occult chest injury. There were 1067 (56%) patients with a negative abdominal exam who had a A/P CT. From these patients, 174 (16%) were found to have an occult injury on CT. In the third grouping, 592 (43%) of the patients with normal C/A/P exams received a C/A/P CT. Of these patients, 150 (25%) demonstrated occult injuries by CT (Tables 1 and 2). In total, 434 patients with a negative CXR also received a chest CT. Out of this grouping, 151 (35%) had injuries detected on the chest CT. The three subgroups and CXR data can be seen in Fig. 1. Itemized injuries are detailed in Tables 3, 4, 5.

**Table 1:** Patient characteristics with and without chest symptoms

	Total sample	Asymptomatic chest	Symptomatic chest
N	2306	1571	735
Age (years)	52.07 ± 22.41	52. 20 ± 22.83	51. 81 ± 21.54
Male (%)	56	56	56
ISS	8.21 ± 6.31	7.51 ± 5.68	9.60 ± 7.25
Hospital LOS	4.02 ± 4.10	4.14 ± 4.33	3.72 ± 3.38
Mortality	0.43%	0.52%	0.28%

*ISS* Injury severity score, *LOS* Length of stay

**Table 2:** Occult injuries detected on CT by body region(s)

Asymptomatic body region	Number of patients	Corresponding CT	Number of positive CT
Chest	1571 (68%)	829 (54%)	127 (15%)
Abdomen	1903 (83%)	1067 (56%)	174 (16%)
C/A/P	1375 (60%)	592 (43%)	150 (25%)

*C/A/P* Chest/abdomen/pelvis, *CT* Computed tomography

**Table 3:** Occult chest injuries

Normal chest PE with abnormal CT C	Number (%)
Bilateral rib fractures	5 (0.6)
Clavicle fracture	12 (1.5)
Lung contusion	17 (2.1)
Pneumothorax	25 (3.1)
1–2 rib fractures	38 (4.7)
3–6 rib fractures	25 (3.1)
> 6 rib fractures	7 (0.87)
Scapular fracture	14 (1.7)
Splenic injury	4 (0.50)
Suspected aortic injury	3 (0.37)
Hemothorax	1 (0.12)
Sternal fracture	6 (0.75)
Total chest injuries	157 (19.5)
Total abnormal CT C	111 (13.8)

*CT C* Computed tomography, chest, *PE* Physical exam

Total number of patients with normal chest PE with CT C = 805

**Table 4:** Occult chest injuries in negative PE and CXR

Normal chest PE and CXR with abnormal CT C	Number (%)
Bilateral rib fractures	3 (1.1)
Clavicle fracture	1 (0.38)
Lung contusion	7 (2.7)
Pneumothorax	6 (2.3)
1–2 rib fractures	10 (3.8)
3–6 rib fractures	12 (4.6)
> 6 rib fractures	2 (0.77)
Scapular fracture	4 (1.5)
Splenic injury	3 (1.1)
Suspected aortic injury	3 (1.1)
Hemothorax	1 (0.38)
Sternal fracture	5 (1.9)
Total chest injuries	57 (21.8)
Total abnormal CT C	41 (15.7)

*CT C* Computed tomography, chest, *CXR* Chest X-ray, *PE* Physical exam

Total number of patients with normal chest PE and CXR with CT C = 261

**Table 5:** Occult abdominal/pelvic injuries

Normal abdominal PE with abnormal CT A/P	Number (%)
Bowel wall thickening	2 (0.15)
Free air	2 (0.15)
Liver I–III	21 (1.6)
Liver IV	5 (0.38)
Liver V	2 (0.15)
Mesenteric stranding	6 (0.45)
Pelvic fracture	235 (17.7)
Renal contusion	1 (0.07)
Renal laceration	4 (0.30)
Spleen I–II	18 (1.35)
Spleen III	6 (0.45)
Spleen IV–V	7 (0.52)
Suspicious small bowel injury	2 (0.15)
Total A/P injuries	311 (23.4)
Total abnormal CT A/P	309 (23.2)
Total abdominal injuries excluding pelvis	76 (5.7)

*CT A/P* Computed tomography of abdomen/pelvis, *PE* Physical exam

Total number of patients with normal A/P PE with CT A/P = 1331

Asymptomatic patients with a negative CT of the chest and/or abdomen and pelvis had a mean length of stay (LOS) 1 day less than asymptomatic patients without a CT of the corresponding body region(s) (*p* < 0.001) (Table 6).

**Table 6:** LOS and ISS in asymptomatic body region with negative CT vs no CT

	Negative CT	No CT	Significance
Chest			
*n* (%)	702 (45)	742 (47)	
LOS	3.45 ± 4.28	4.61 ± 4.14	*p* < 0.001
ISS	7.35 ± 5.60	6.70 ± 4.73	*p* = 0.017
A/P			
*n* (%)	893 (47)	836 (44)	
LOS	3.36 ± 4.07	4.38 ± 3.96	*p* < 0.001
ISS	8.30 ± 6.57	7.01 ± 4.74	*p* < 0.001
C/A/P			
*n* (%)	442 (32)	783 (57)	
LOS	3.29 ± 4.56	4.53 ± 4.11	*p* < 0.001
ISS	7.83 ± 6.07	6.77 ± 4.61	*p* < 0.001

*A/P* Abdomen/pelvis, *C/A/P* Chest/abdomen/pelvis, *CT* Computed tomography, *ISS* Injury Severity Score, *LOS* Length of Stay

Values are means ± standard deviations

There were 10 deaths (0.43%) in this cohort. There was no difference in mortality between asymptomatic chest with CT C and without CT C (4 (0.3%) vs 4 (0.3%), *p* = 0.575) or asymptomatic C/A/P with CT C/A/P or without (2 (0.1%) vs 4 (0.3%), *p* = 0.481). There was a statistical significance in mortality between those with asymptomatic abdominal exam with CT A/P and without (2 (0.1%) vs 7 (0.4%), *p* = 0.043) (Table 7). No incidents of contrast-induced complications were noted in the study period.

**Table 7:** Mortality in asymptomatic body region with and without CT

Body region	Corresponding CT	No corresponding CT	*p*
Chest	4 (0.3%)	4 (0.3%)	0.575
Abdomen	2 (0.1%)	7 (0.4%)	0.043
Chest/abd/pelvis	2 (0.1%)	4 (0.3%)	0.481

*Abd* Abdomen, *CT* Computed tomography

There were 2 asymptomatic patients that did not initially receive a CT, but later did. One revealed a hemothorax, 3 rib fractures, and a left diaphragmatic hernia. Another patient was found to have a grade 3 liver laceration. No other patients were recorded that presented with an asymptomatic body region exam without initial CT that was later found to have an occult injury on delayed CT. There were 10 delayed minor injuries recorded after radiologist overread.

## Discussion

It is not surprising that overall mortality is low (0.43%) in this patient cohort given the low ISS. Even though we found a statistical significance in mortality between asymptomatic abdominal region with and without CT A/P, the numbers are low, and it is difficult to state that there is a real clinical benefit here.

We are not able to prove a benefit in morbidity for these patients based on the data. Only 2 patients that had no chest or abdominal findings on physical exam and did not have an initial torso CT were found to have injuries on a delayed torso CT. This is consistent with a Cochrane review by Van Vugt et al. published in 2013 comparing selective torso CT versus routine torso CT—there just have not been enough quality trials to base a recommendation [18].

One year later, Caputo et al. published a systematic review and meta-analysis on whole-body CT versus selective CT in trauma patients that did show a significant mortality benefit for those that receive pan CT, even though their ISS was higher [24]. This study is different in the fact that Caputo looked at whole-body CT and the Cochrane review was specifically thoracoabdominal CT, as in our study.

Although they are not randomized controlled trials, there are several studies supporting pan CT in trauma. Salim et al. reported findings in a prospective observational study that changed management in 19% of stable trauma patients that received pan CT [8]. Yeguiayan et al. showed a 30-day reduction in mortality from 22% to 16% by using pan CT, and Self et al. showed that 26% of patients receiving CT C/A/P who were already receiving a head CT had unexpected findings that changed treatment [16, 25].

The first multicenter, randomized controlled trial (REACT-2) conducted by Sierink et al. compared immediate total-body CT with conventional imaging and selective CT. The authors concluded that immediate total-body CT was safer, quicker, and does not increase direct medical costs. However, they also found that this imaging does not change in-hospital mortality [26]. The median ISS (20) was significantly higher than in our cohort, so we do not have a direct comparison with that study group.

The study by Lee et al. comparing the cost-effectiveness of pan CT versus selective CT in stable, young adults resembles our cohort. The average ISS was 5 in this study, compared to 8 in ours and their population was much more uniform. They concluded that it is cost-effective to use pan CT based on mechanism alone, even in these mildly injured patients [20]. To relate cost-effectiveness to this study, the price for a CT A/P and the reading of it by a radiologist would be approximately $1200 at our hospital, whereas a single day in the Trauma/Neurology Intensive care unit (TNICU) is approximately $6500 and a day in the orthopedic unit is almost $3000. This difference in price is significant and therefore it should be recognized that this reduction in LOS is cost-effective for the patient. It is noted that the cost of incidentalomas and contrast-induced complications were not included in that study [20]. However, one should consider the benefits of serendipitous early detection of malignancy. We do not have data to show for this beyond personal experience and is perhaps a future area to study.

The risk of radiation exposure is always a concern with CT imaging. Sierink et al. published a study in 2013 showing an increase in initial radiation exposure after instituting a total-body CT protocol, but that total in-hospital radiation exposure was similar [27]. Another study also showed an increase in patients receiving more radiation (> 20 mSv) after instituting a trauma pan scan protocol [28]. The REACT-2 trial only showed a 0.3 mSv difference (i.e., 1 CXR) in radiation exposure of pan scan vs selectively scanned trauma patients. The exposure to radiation during a CT scan is easy to establish, but to say the risk of cancer conferred by that exposure is extrapolated and may not be accurate; however, best estimates are about 29,000 cases of cancer are attributed to CT scans in the United States annually [29]. Tien et al. published a prospective cohort study of trauma patients’ average radiation exposure of 22.7 mSv level, which would be estimated to result in 190 cancer-related deaths per 100,000 patients exposed [30]. Though still greatly debated, it is our opinion that a single torso CT benefits outweigh this relatively small, theoretical risk in adults.

We show, in our retrospective study of mildly injured blunt trauma patients with a GCS of 14 or 15, that a surprising number of injuries are detected after normal chest and abdominal physical examinations, as well as chest X-ray. Four hundred sixty-eight injuries (or signs of suspected injuries requiring a change in management) were detected in 420 otherwise asymptomatic, evaluable patients. This is 14% of the patients with benign chest exam and 23% with benign abdominal bedside exam findings. One hundered fifty of 592 (25%) patients with a complete benign exam of the torso ended up having occult injuries on CT C/A/P. The known lack of sensitivity of CXR is consistent in our study (61%).

Whether these occult findings are clinically relevant is an important point. Some may argue that clinical importance is only if a procedure is performed or if an early discharge is accomplished. We found the length of stay of a patient receiving negative torso CT was 1 day less than similar patients that did not receive torso CT. Additionally, stratifying patients to level of care (floor versus intensive care unit) has been consistently shown to be important, especially with regard to the number of ribs fractured, even in patients as young as 45 [31, 32]. There is also data that supports significant post-hospital morbidity exists for patients after relatively minor thoracic trauma [33, 34]. Having the knowledge of the full extent of injury may be important in post-discharge rehabilitation plans and expectations.

In addition, quicker diagnosis leads to shorter wait time to intervention when needed. Reporting a negative torso CT is reassuring for both the patient and physician, as well as leading to the shorter hospital length of stay as found in our study.

The limitations of this study include its retrospective nature and lack of cost analysis. The blunt mechanisms were also not stratified based on the height of fall, motor vehicle rollover or ejection, etc. which hinders further and more specific stratification. Various trauma laboratory testing was not recorded as many of the patients were trauma alerts or nonactivations, which often do not have full laboratory testing conducted (i.e., arterial blood gas, urine drug screen). Focused assessment with sonography for trauma (FAST) exams were not consistently performed in this cohort and therefore were not analyzed. FAST exams are generally used in higher acuity patients in shock and performed by trained ER physicians; however, these exams are not used liberally at our institution during this time. Additionally, these were not E-FAST exams and did not include sonography of the chest. Perhaps if these above data points were consistently performed and recorded, a more specific patient population subset could be identified that would better predict the need for cross-sectional imaging of the torso. Finally, there is one concern in this study. The initial indication for CT scanning is variable, as it up to the discretion of the ED physician (based upon physical examination without clear guidelines). This variability means the study must be interpreted with caution, as it is susceptible to bias.

## Conclusion

A significant number of occult injuries were detected in stable adult blunt trauma patients with a GCS of 14/15. A negative physical exam combined with a normal CXR does not rule out the presence of occult injuries and the need for torso imaging. In blunt trauma patients with normal sensorium, physical exam, and CXR, the practice of obtaining cross-sectional imaging would appear to be beneficial by increasing the accuracy of total injury burden and decreasing hospital length of stay. These benefits outweigh the small risk associated with CT scan.

## Data Availability

The datasets used and/or analyzed during the current study are available from the corresponding author on reasonable request.

## Abbreviations

Abd: Abdomen/abdominal
ATLS: Advanced trauma life support
C/A/P: Chest/abdomen/pelvis
CT: Computed tomography
CW: Chest wall
CXR: Chest X-ray
ED: Emergency Department
FAST: Focused assessment with sonography for trauma
GCS: Glasgow Coma Scale
iCT: Initial computed tomography
INR: International normalized ratio
ISS: Injury Severity Score
MRI: Magnetic resonance imaging
Pan CT: Computed tomography of the head, cervical spine, chest, abdomen, and pelvis
PE: Physical examination
PTX: Pneumothorax
TNICU: Trauma/Neurology Intensive Care Unit
